# Imaging intensive care patients: multidisciplinary conferences as a quality improvement initiative to reduce medical error

**DOI:** 10.1186/s13244-022-01313-5

**Published:** 2022-11-04

**Authors:** Gloria Muench, Denis Witham, Kerstin Rubarth, Elke Zimmermann, Susanne Marz, Damaris Praeger, Viktor Wegener, Jens Nee, Marc Dewey, Julian Pohlan

**Affiliations:** 1 Department of Radiology, Campus Charité Mitte, Charité – Universitätsmedizin Berlin, Freie Universität Berlin, Humboldt-Universität zu Berlin, and Berlin Institute of Health, Charitéplatz 1, 10117 Berlin, Germany; 2 Department of Cardiology, Campus Charité Mitte, Charité – Universitätsmedizin Berlin, Freie Universität Berlin, Humboldt-Universität zu Berlin, and Berlin Institute of Health, Charitéplatz 1, 10117 Berlin, Germany; 3 Institute of Biometry and Clinical Epidemiology, Charité - Universitätsmedizin Berlin, Freie Universität Berlin and Humboldt-Universität zu Berlin, Charitéplatz 1, 10117 Berlin, Germany; 4 Berlin Institute of Health (BIH), Anna-Louisa-Karsch-Straße 2, 10178 Berlin, Germany; 5 Department of Surgery with Intensive Care, Campus Charité Mitte, Charité – Universitätsmedizin Berlin, Freie Universität Berlin, Humboldt-Universität zu Berlin, and Berlin Institute of Health, Charitéplatz 1, 10117 Berlin, Germany; 6 Department of Anesthesiology and Operative Intensive Care Medicine (CCM, CVK), Charité - Universitätsmedizin Berlin, Freie Universität Berlin, Humboldt-Universität zu Berlin, and Berlin Institute of Health, Berlin, Germany; 7 Department of Nephrology and intensive care, Campus Charité Mitte, Charité – Universitätsmedizin Berlin, Freie Universitäts Berlin and Humboldt Universität zu Berlin, Charitéplatz 1, 10117 Berlin, Germany

**Keywords:** Quality improvement, Intensive care units, Critical care, Interdisciplinary communication, Feedback

## Abstract

**Background:**

Strategies to identify imaging-related error and minimise its consequences are important in the management of critically ill patients. A new quality management (QM) initiative for radiological examinations has been implemented in an intensive care unit (ICU) setting. In regular multidisciplinary conferences (MDCs), radiologists and ICU physicians re-evaluate recent examinations. Structured bilateral feedback is provided to identify errors early. This study aims at investigating its impact on the occurrence of QM events (imaging-related errors). Standardised protocols of all MDCs from 1st of June 2018 through 31st of December 2019 were analysed with regard to categories of QM events (i.e. indication, procedure, report) and resulting consequences.

**Results:**

We analysed 241 MDCs with a total of 973 examinations. 14.0% (*n* = 136/973) of examinations were affected by QM events. The majority of events were report-related (76.3%, *n* = 106/139, e.g. misinterpreted finding), followed by procedure-related (18.0%, *n* = 25/139, e.g. technical issue) and indication-related events (5.8%, *n* = 8/139, e.g. faulty indication). The median time until identification of a QM event (time to MDC) was 2 days (interquartile range = 2). Comparing the first to the second half of the intervention period, the incidence of QM events decreased significantly from 22.9% (*n* = 109/476) to 6.0% (*n* = 30/497) (*p* < 0.0001). Significance of this effect was confirmed by linear regression (*p* < 0.0001).

**Conclusions:**

Establishing structured discussion and feedback between radiologists and intensive care physicians in the form of MDCs is associated with a statistically significant reduction in QM events. These results indicate that MDCs may be one suitable approach to timely identify imaging-related error.

**Supplementary Information:**

The online version contains supplementary material available at 10.1186/s13244-022-01313-5.

## Key points

- Multidisciplinary conferences (MDCs) between radiologists and intensive care physicians improve feedback.
- MDCs allow timely identification of quality management (QM) events (imaging-related errors).
- The incidence of QM events significantly decreased after implementation of MDCs.

## Introduction

Imaging plays an integral role in diagnostic workup, but the interpretation varies inter-individually. Seemingly minor errors at this stage may have serious consequences for patients. Thus, it is of great urgency to refine work processes to reduce the likelihood of errors and to identify them before leading to harm [1]. While structured feedback from senior to junior radiologists is part of clinical routine in our and many other departments, a specific way for interdisciplinary feedback between clinicians and radiologists was not available for patients from intensive care units (ICUs) in the past. ICU patients suffer from complex conditions and require management by a multidisciplinary team, which in turn requires structured communication.

Error prevention and safety event reporting in radiology has been investigated before. Garland, a pioneer in the field of diagnostic accuracy studies, published error rates of around 30% in the 1950ies/60ies, and more recent studies suggest that the situation has improved only incrementally during time [2–6]. Kim et al. retrospectively analysed radiological examinations and identified findings initially overlooked. The time between the initial imaging examination, when the finding was misinterpreted and its detection was 251 days on average [7]. These results underline the importance of integrating regular structured feedback into clinical routine in order to quickly identify any diagnostic errors before patients are harmed, which, to the best of our knowledge, has been attempted only by a few quality initiatives [8]. For example, systematic and independent second reading to improve patient safety is standardly performed in mammography with varying degree of statistical evidence for this rationale [9]. Several other studies also focussed on the quality of radiological reports [10–16]. Indication and the examination procedure itself may also be flawed. There is very little data on the contribution of these factors to diagnostic error [14]. In a focussed group discussion, there was agreement among radiologists that inadequate request forms pose a significant problem for diagnostic quality and that regular interdisciplinary discussion with clinicians is necessary to ensure effective diagnostic processes [17]. An analysis of the consequences following informal consultations between clinicians and radiologists revealed changes to the report in one quarter of documented cases [18]. Clinicians appreciate face-to-face contact for direct interprofessional exchange with radiologists [19]. Still, without implementation of formal institutional work processes, reluctance to voice a concern poses a high reporting threshold: More than 50% of radiologists self-reported to not always speaking up about safety concerns [20]. “Providing continued feedback regarding safety reporting “ could lower this barrier by establishing a routine [21].

In 2018, alongside the opening of two new intensive care units (ICUs), we implemented a new systematic QM tool in our department. Regular multidisciplinary conferences (MDCs) involving both, intensive care physicians, and radiologists were introduced. These conferences follow the principles of any radiological demonstration: radiologists present and re-interpret radiological examinations on the grounds of clinical information potentially unavailable to the first reader. In contrast to normal radiological demonstrations, the here evaluated MDCs differ in their structure, the immediate option for bilateral feedback and a systematic documentation of the discussion. This study aims at analysing how these MDCs affect the incidence of QM events (imaging-related errors) over time.

## Methods

### Quality management initiative

MDCs take place twice a week, separately for the medical and surgical ICU and are attended by radiologists and ICU physicians and optionally by other interested parties. ICU physicians include internal medicine specialists for the medical ICU and both, anaesthesiologists, and surgeons for the surgical ICU. Patients to be discussed are chosen on an individual basis by the treating ICU physicians. During the time investigated here, they were unaware about an ongoing study on the MDC. Usually, cases are chosen due to their complexity, unclear clinical presentation or uncertain radiological findings. Multiple examinations of the same patient may be enrolled in the same MDC. QM events (imaging-related errors) are specific to a certain examination (i.e. in patients with multiple enrolled examinations). MDCs are hosted by the radiology department. Two senior board-certified radiologists take turn as chairs to ensure consistency. Both have more than 15 years of experience in radiology with expertise in emergency and ICU imaging. All MDCs follow the same structure: basic patient information, clinical aspects, presentation of radiological findings, discussion of clinical relevance and therapeutic consequences with special focus on ambiguities, identification of QM events (imaging-related errors), feedback. Clinical aspects (including all clinical data, lab results and microbiological testings) are available during the MDC and constitute the base for further discussion and interpretation of radiological findings. A standardised protocol consisting of two sections includes the following information: patient name, date and type of examination, as well as body region in hand with a clinical question in section A; occurrence and type of QM event (a definition of which is given below) and an option for further handwritten comments in section B (Additional file 1: Fig. 1). Section A is filled out by the ICU physicians and sent to the radiology department prior to each conference, allowing for adequate preparation. Section B is filled out by the leading radiologist in consultation with all physicians attending the conference. QM events are any imaging-related errors and are defined as falling into one of the following categories: incidents related to indication, procedure, or reporting (examples of each category are provided in Additional file 1: Table 1). Reported events are described in the comment section and include any deviation from departmental or hospital guidelines. The exact classification of such an incident as a QM events is left to the case-specific interpretation of all attendees. The content of the written comments is related mainly to the respective QM event and includes a more detailed description of the nature of the incident. Some comments also provide general feedback on both the radiological workup process and the conduct of the conferences. All attendees, regardless of their rank, are invited to both provide and receive feedback. If applicable, specific feedback in the form of phone calls or e-mails is given to non-attending physicians involved in a QM event.

### Subjects

**Fig. 1:**
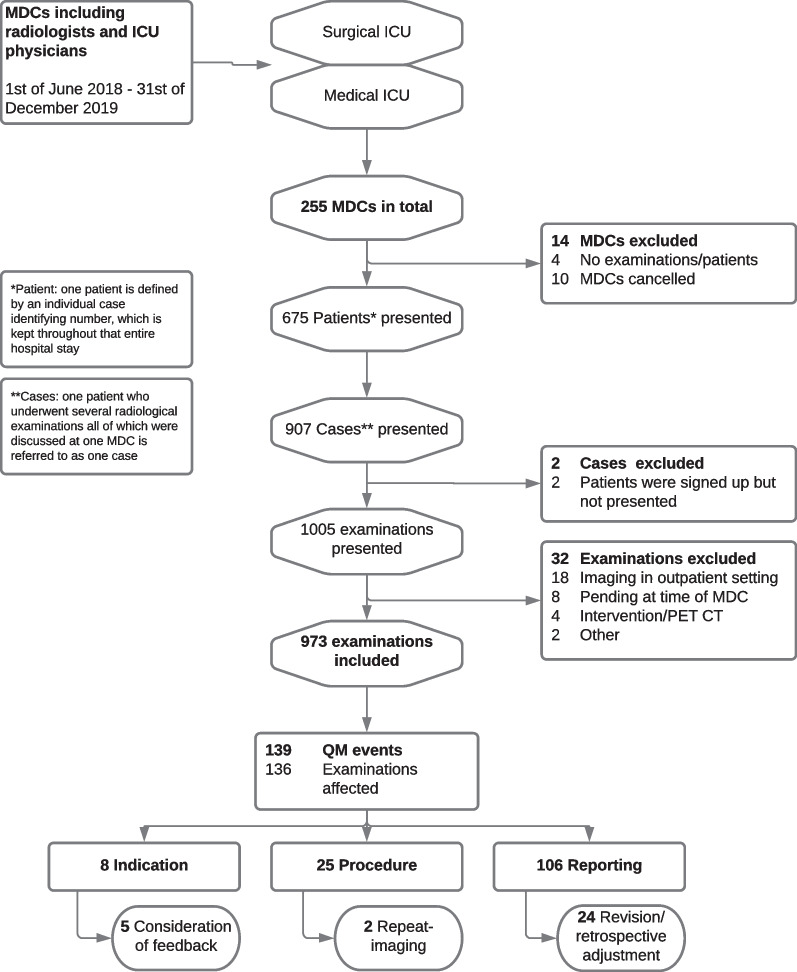
Patient flow chart. Multidisciplinary conferences (MDC) for a medical and for a surgical intensive care unit (ICU) were introduced at this university hospital in 2018. The MDCs take place twice a week separately for the medical and surgical ICU. Radiologists present recent imaging studies which are then re-interpreted and discussed a second time with the treating ICU physicians. Bilateral feedback is provided and the occurrence of QM events is documented. Standardised protocols of all scheduled MDCs were collected over a 19-month period and analysed with regard to different QM categories and QM consequences. For some days, no MDCs were scheduled due to public holidays (*n* = 10) or organisational reasons (*n* = 63)

This is a retrospectively conducted observational analytic study. All scheduled MDCs during the study period were protocolled and included in this analysis. Scheduled MDCs that were cancelled or for which no examinations were registered were subsequently excluded from the analysis. All patients who underwent at least one radiological examination discussed during an MDC were included. Radiological interventions like invasive angiographies and integrated external radiological examinations were excluded from this analysis. Patients with several imaging examinations fulfilling our inclusion criteria were enrolled multiple times. In cases where patients underwent different imaging examinations that were discussed in the same conference, each examination was regarded as a separate exam (see Fig. 1: Patient flow chart). Examinations were further classified according to the body region examined. Examinations of several body regions (e.g. a whole-body CT scan) were included in each of the body region categories covered: e.g. head, chest, abdomen, other (Table 1). Demographics of the patient population are summarised in Table 2. This study was approved by the local ethics committee under the number EA1/024/21. Retrospective consent from patients was waived. The Declaration of Helsinki was respected.

**Table 1:** Radiological examinations

	Number	Percentage (%)
*Imaging modality*		
CT	577	88.6
MRI	34	5.2
X-ray	40	6.1
*Body region examined*		
Head	128	40.5
Neck	25	7.9
Chest	188	59.5
Abdomen	129	40.8
Pelvis	95	30.1
Other	12	3.8

Each category of body region comprises all examinations performed of that region. E.g. a CT head, abdomen, legs is included in all of those categories: head, abdomen, other. “Other” includes examinations of the spine (*n* = 5), heart (*n* = 4), and leg(s) (*n* = 3). Percentages were calculated for examinations in which imaging modality (*n* = 651) or body region examined (*n* = 316) were documented. In 322 examinations, imaging modality was unknown and in 657 examinations, body region examined was unknown

**Table 2:** Patient characteristics

Variable		Number	Percentage
No of patients		675	
Sex	Male	571	58.7%
	Female	381	39.2%
	Unknown	21	
Age mean (standard deviation (SD))	Total	61.4 years (± 17.5)	
	Male	61.8 years	
	Female	61.1 years	
Time to MDC	Median (interquartile range (IQR))	2 days (2)	
Examinations included		973	
Examinations/MDC	Median (IQR)	4 (2)	

Sex and time to multidisciplinary conference (MDC) were documented for individual examinations (*n* = 973). Patients who underwent multiple examinations discussed at one/several MDCs were enrolled for each of those examinations. Time to MDC was defined as the time between examination and MDC during which the examination was discussed

### Data collection

Handwritten reports from the medical and surgical ICU at one university hospital were collected retrospectively over a 19-month period from 1st of June 2018 through 31st of December 2019. All reports were digitalised by one doctoral candidate using *Microsoft® Office Excel 2016*. Data protection was guaranteed. All QM events were looked up in the patient management system (*SAP® Software 2021 SAP SE or an SAP affiliate company*) and radiological information system *(Centricity™ RIS-I 6 2018 General Electric Company)* to extract further information regarding the examinations. Any consequences from QM events documented in the protocols or from medical records were extracted and categorised as displayed in Table 3.

**Table 3:** Quality management (QM) consequences

QM category	QM consequence	Number	Percentage (%)
Indication		8	5.8
	Consideration of feedback	5	62.5
	None apparent	2	25.0
	Untraceable	1	12.5
Procedure		25	18.0
	Repeat imaging	2	8.0
	None apparent	11	44.0
	Untraceable	12	48.0
Reporting		106	76.3
	Addendum to report	17	16.0
	Revision/retrospective adjustment	7	6.6
	None apparent (incl. persisting discrepancy)	17	16.0
	Untraceable	65	61.3

All examinations affected by QM events were retrieved from the patient record, and any consequences attributable to these QM events were documented. Consideration of feedback applies to indication-related QM events, e.g. request forms that were criticised for containing insufficient detail were followed by detailed and precise request forms for the next examinations. Repeat imaging concerns procedural QM events, after which patients underwent repeat-imaging following the correct protocol. Addendum to report refers to the creation of a separate report including the initially overlooked/misinterpreted finding, which was added to the original report. In revised/retrospectively adjusted reports, the original reports were edited to include the newly discovered/altered findings

### Statistical analysis

*Excel®* and *SPSS®* were used for statistical analyses. Mean and standard deviation are provided for descriptive statistics. If normal distribution was not given, median and interquartile range are provided. Chi-Square tests were employed to compare the incidence of QM events between the first and second half of the intervention period and between different QM categories. Data were analysed in terms of correlation and linearity. The effect of time (increasing number of previously conducted MDCs) as a single predictor of the incidence of QM events was calculated. Hence, simple linear regression analysis was performed to assess the incidence of QM events over time after the implementation of the new QM tool. A *p* value < 0.05 was considered to be statistically significant. Due to the exploratory characteristic of the study, no adjustment for multiplicity was applied.

## Results

### Basic characteristics

Our analysis included 241 MDCs. One hundred and twenty-seven were attributed to a medical ward and 114 to a surgical ward. A total of 1,005 radiological examinations were presented with a median of 4 (interquartile range (IQR) = 2) examinations per MDC. The median time between an examination and its discussion in an MDC (time to MDC) was 2 days (IQR = 2). A total of 60 patients were enrolled in an MDC with multiple examinations. In 34 out of 60 examinations were performed on different days. The most frequently asked clinical question regarded infectious foci, followed by bleeding and tumours in descending order.

### Quality management events

**Fig. 2:**
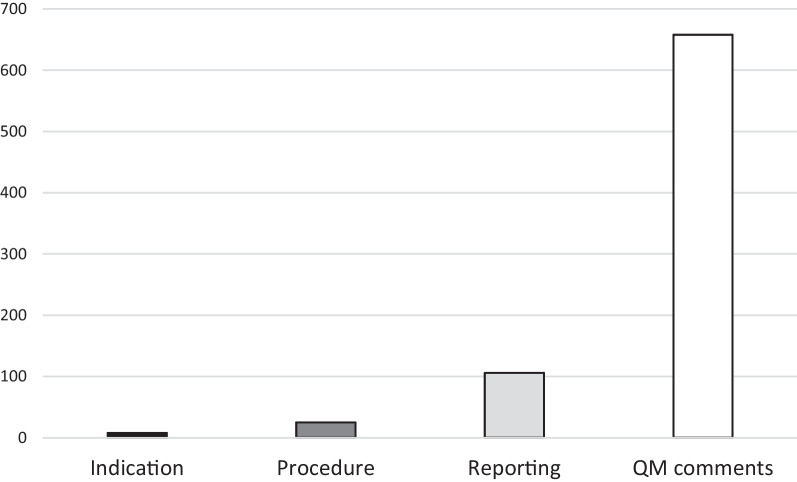
QM events by category. QM events were categorised into incidents related to indication (*n* = 8, 5.8%), procedure (*n* = 25, 18.0%), and reporting (*n* = 106, 76.3%). The standardised protocol used for documentation of MDCs has a box for entering free text (QM comments), which was used in 658 (67.6%) of all examinations presented in an MDC (*n* = 973). In examinations affected by QM events, QM comments further addressed and explained the respective event. In examinations not affected by QM events, QM comments provided general feedback for further improvement

A total of 139 imaging-related QM events were reported during the study period (1st of June 2018 through 31st of December 2019). QM events occurred in 136 examinations (14.0%), resulting in an incidence of 0.53 QM events per MDC, i.e. during every second MDC one QM was reported. No repeat errors (i.e. multiple QM events affecting the same aspect in the same patient) occurred. Most QM events were related to the radiological report (*n* = 106), followed by procedure (*n* = 25), and indication (*n* = 8) (details are given in Table 4). No statistically significant difference in the occurrence of QM events was calculated for the different QM categories (*p* = 0.941). In 658 examinations, an additional comment was noted during the MDC (Fig. 2), mostly related to the respective QM event. In 533 examinations, a comment with general feedback was provided even if no QM event occurred or the comment did not solely refer to the respective QM event but provided further feedback.

**Table 4:** Quality management (QM) events

QM category	Number	Percentage of total QM events (*n* = 139)	Percentage of examinations affected (*n* = 973)
Indication	8	5.8%	0.8%
Procedure	25	18.0%	2.6%
Reporting	106	76.3%	10.9%

Incidence of QM events by category

### Consequences of QM events

All examinations affected by QM events were retrieved from the patient record and any consequences attributable to these QM events were documented (Table 3). Indication-related QM events lead to a consideration of the feedback i.e. an improvement of the following request forms in 62.5% (*n* = 5/8) of cases. Procedure-related events were followed by repeat imaging in 8.0% (*n* = 2/25). In 22.6% of examinations affected by reporting-related events, the report was retrospectively revised or adjusted (*n* = 7/106, 6.6%) or an addendum was written (*n* = 17/106, 16.0%). In most cases (*n* = 108/139, 77.7%), no consequence was apparent or possible consequences could not definitely be linked to the respective QM event.

### QM events over time

**Fig. 3:**
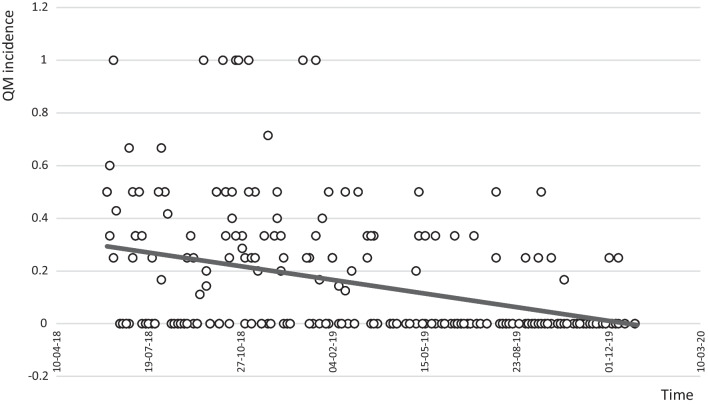
Incidence of QM events over time. Each dot represents one MDC at a specific time of the intervention period. The QM incidence (Y-axis) shows the number of QM events per examination presented for each of those MDCs. QM events decreased significantly by 73.6% from the first (*n* = 109/476) to the second half (*n* = 30/497) of the intervention period (*p* < 0.0001) as reflected by the trend line. This significant decrease in QM events over time was confirmed by linear regression (*p* = 0.0001) after testing for respective requirements

The number of QM events per MDCs decreased over time during the intervention period (Fig. 3): a statistically significant decrease of 73.6% in the incidence of QM events from the first to the second half of the intervention period was calculated (*p* < 0.0001). I.e. in the first half, 22.9% (*n* = 109/476) of examinations were affected by QM events and 6.0% (*n* = 30/497) in the second half. At the same time, the number of examinations per MDC remained constant (Additional file 1: Fig. 2). Simple linear regression analysis yielded a statistically significant effect of time on the proportion of QM events per examination discussed (regression coefficient estimate = −0.001, 95% confidence interval: [−0.002, −0.001], *p* < 0.0001). The model with time as a single predictor accounts for 13.5% of the variance in the incidence of QM events (*R*^2^ = 0.135; further statistical results are provided in Additional file 1: Table 2).

## Discussion

### Summary

The total incidence of QM events in the radiological workup process of intensive care patients decreased over time with statistical significance after the implementation of a new quality management initiative consisting of regular multidisciplinary conferences with a direct feedback mechanism between radiologists and ICU physicians. The majority of imaging-related QM events in our analysis affected the radiological report. The most frequent consequence was a revision or retrospective adjustment of the respective report. A standardised protocol for providing written feedback was frequently made use of.

### Interpretation

Structured bilateral feedback is associated with a decline of imaging-related adverse events for patients. The implementation of an effective mechanism for timely identification of errors may contain their detrimental effects.

### Comparison with the literature

Dedicated investigation of QM events confirms the hypothesis that unsafe acts appear in recurrent patterns rather than being individual mistakes [22]. Hence, a systematic approach to combat the risk of error on an organisational level as presented here is crucial to ensure patient safety. The introduction of reporting systems was highly recommended by the Committee on Quality of Health Care in America [1]. In a more recent report, the committee further encouraged a greater focus on patient-centredness and effectiveness, both of which were addressed by this QM initiative [23]. The integration of both, clinical, and radiological aspects within a multidisciplinary team allows for a more holistic patient view and therefore a more personalised patient management as special attention is paid to each patient’s current condition rather than simply their diagnoses. The error rate we found is lower than reported by other investigators [5, 6, 24, 25]. One explanation might be that imaging examinations are routinely interpreted by two readers in our department. This standardised double reading was not mentioned in the studies above. Most published diagnostic error studies retrospectively identified adverse events over a certain time period. Conversely, our approach was to integrate both the detection of errors and the implementation of a constant feedback loop into clinical routine. An effect in terms of reducing QM event rates appears to have occurred early after implementation. Our results suggest that, regarding imaging-related errors, the radiological report is especially prone to error. This is in accordance with published studies, which focus mainly on the radiological report as a source of adverse events [10–15]. Tracking radiologists’ eye movements during the reading of verified chest imaging studies showed that they spent longer time dwelling on overlooked findings [26]. If a finding is initially detected, but wrongly interpreted as insignificant, retrospective multidisciplinary discussion possibly allows for interpretation improvements as agreement within the teams is reached. Our study population included more male than female patients (60% versus 40%), which reflects the usual sex distribution of ICU patients in many countries [27–30]. The high number of CTs in comparison to X-rays presented in MDCs may be explained by their higher complexity both in clinical questions to be answered and their interpretation. Hence, there may be more need for multidisciplinary consultation. Additionally, ICU physicians may request a higher-resolution imaging modality in case of inconclusive X-ray findings before registering the patient for an MDC. We have previously investigated the use of CT for the identification of septic foci and in doing so, have found that unclear infectious sources are associated with low reader confidence [31]. This may explain why most registered examinations focussed on infectious source identification, often concerning septic patients. Systematic error identification may therefore be especially important in septic patients.

### Clinical impact

While error eradication seems impossible, error prevention and reduction are still worth pursuing. Another aim should be to identify imaging-related QM events before they cause harm. Correcting errors by early reporting may impede adverse events in patients. Establishing a regular structured feedback mechanism should benefit all participants and may thus reduce errors as well as improve the overall quality of the diagnostic workup process. Furthermore, an established feedback culture creates an environment encouraging everyone, especially junior physicians, to speak up more. With declining QM event rates, the structured collection of written feedback during MDCs might still prove important for maintaining awareness of quality control in clinical routine.

### Limitations

The retrospective design of this study results in several limitations. For example, comparison between pre- and post-interventional periods was not possible. The documentation of QM events, the dependent variable, was part of the intervention itself and had thus not been performed previously. To some extent, the observed decline in QM events may be due to learning effects. Importantly, both variable detail of the description in the MDC protocols and handwritten notes rendered the retrieval of some information challenging. Moreover, analysis of errors and related consequences based on medical records alone is limited, especially in ICU patients with complex conditions and is further limited by German data protection law. Our analysis therefore primarily focussed on measurable imaging-related errors. Besides, the time period analysed does not account for the emergence of the COVID-19 pandemic. Its possible impact is being investigated in an ongoing analysis and not within the scope of this manuscript.

## Conclusion

To conclude, the implementation of a bilateral feedback mechanism between radiologists and ICU physicians is associated with a decrease in imaging-related QM events over time. Thus, multidisciplinary re-evaluation of diagnostic results can be seen as a good tool for timely identification of errors. Further research should elucidate the consequences of QM events in more detail.

## Supplementary Information

**Additional file 1:** Supplementary tables and figures.

## Data Availability

The datasets used and analysed during the current study are available from the corresponding author on reasonable request.

## Abbreviations

ICU: Intensive care unit
MDC: Multidisciplinary conference
QM: Quality management
