# Supplementary material for: Imaging intensive care patients: multidisciplinary conferences as a quality improvement initiative to reduce medical error
Source: Insights Imaging. 2022 Nov 4;13:175. doi: 10.1186/s13244-022-01313-5 (PMC9636350; doi:10.1186/s13244-022-01313-5)

ELECTRONIC SUPPLEMENTARY MATERIAL

Imaging intensive care patients: multidisciplinary conferences as a quality improvement initiative to reduce medical error

Supplementary table 1: Examples of QM events.

QM events detected in MDCs were further classified by category. The standardised protocol employed for documentation includes a box for free text comments for further explanation of the respective QM event.

QM category	Responsibility	QM event	Comment
**Indication**	Clinicians	Faulty indication	“No indication for MRI of spine”
		Request form lacking substantial information	“Where? Left/right? Front/back?”
**Procedure**	Radiologists	Failure to administer contrast agent	“cCT also with contrast”
		Technical issues	“Procedure according to resuscitation protocol: venous upper abdomen; due to technical problems without contrast”
**Report**	Radiologists	Misinterpreted finding	“The above-mentioned tumorous lesions in the true pelvis are most likely consistent with uterine fibroids”
		Overlooked finding	“Free air -> perforation of colon”

Supplementary table 2: Summary output regression statistics.

A negative linear correlation between time and QM incidence was found. Simple linear regression was performed to calculate the suitability of time *(increasing number of previously conducted MDCs)* as an individual predictor of the proportion of QM events per examination presented. The result was statistically significant. Hence, the incidence of QM events decreased significantly in the course of the intervention period as the number of prior MDCs increased.

Regression statistics

	R	*R^2*	*Std. error of the estimate*			
	0.363	*0.132*	*0.223*			
ANOVA						
	Sum of squares	*df*	*Mean square*	*F*	*P-value F*	
Regression	*1.799*	*1*	*1.799*	*36.268*	*<0.0001*	
Residual	*11.856*	*239*	*0.050*			
Total	*13.655*	*240*				
Coefficients						
	Unstandardised coefficients				*95% confidence interval for B*	
	Coefficient B	*Standard error*	*T*	*P-value*	*Lower bounds*	*Upper bounds*
Constant	*0.302*	*0.029*	*10.480*	*<0.0001*	*0.245*	*0.358*
MDC	*-0.001*	*<0.0001*	*-6.022*	*<0.0001*	*-0.002*	*-0.001*

Supplementary figure 1

MDC protocol.

This standardised protocol was employed for documentation of all MDCs. The columns on the left side (Section A: Patient information, Examination + date, Clinical question) are filled out by the clinicians in advance and are sent to the radiology department prior to each MDC, allowing radiologist to prepare case presentations. The columns on the right side (Section B: Quality management events, Additional comments; highlighted in gray) are filled out by the radiologist in charge during the MDC. The definition of a QM event is left to attending physicians and jointly agreed upon by the interdisciplinary team.

ICU *X* – Multidisciplinary conference – *HH:MM h* – *Room XX* – *DD/MM/YY*

Patient Information	Examination + Date	Clinical question	Quality management events			Additional comments
			Indication	Procedure	Report	
SURNAME, NAME, SEX<br>*DATE OF BIRTH*<br>*CASE NUMBER*	*IMAGING MODALITY*<br>*DATE OF EXAMINATION*	*From request form, possibly additional aspects*	*X*	*X*	*X*	*-Explanation of QM events*<br>*-Further feedback*

To be filled out and sent to the radiology department (*contact details*) by *XX:XXh*

Supplementary figure 2

Examinations per MDC.

The number of examinations (Y-axis) discussed at each MDC remained constant over time (X-axis).

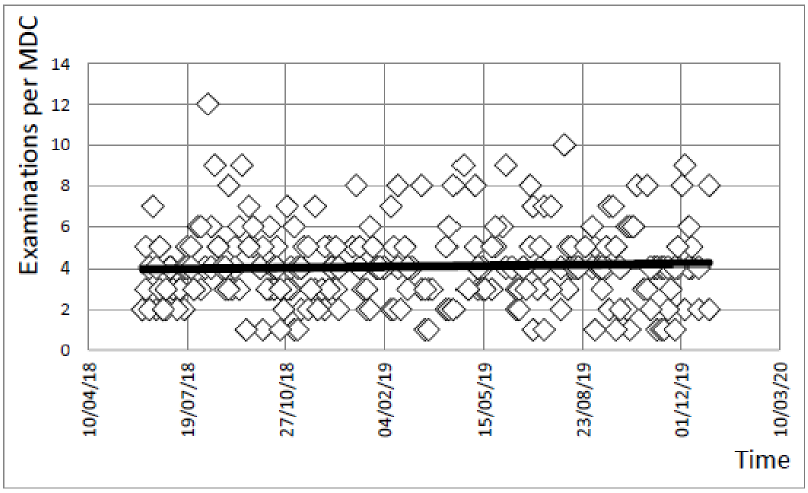

Supplement: Supplementary file 1 — Additional file 1: Supplementary tables and figures. [file 13244_2022_1313_MOESM1_ESM.pdf]
